# Motivations and perceptions of community advisory boards in the ethics of medical research: the case of the Thai-Myanmar border

**DOI:** 10.1186/1472-6939-15-12

**Published:** 2014-02-17

**Authors:** Khin Maung Lwin, Phaik Yeong Cheah, Phaik Kin Cheah, Nicholas J White, Nicholas P J Day, Francois Nosten, Michael Parker

**Affiliations:** 1 Shoklo Malaria Research Unit, Mae Sot, Tak 63110, Thailand; 2 Mahidol Oxford Research Unit, Faculty of Tropical Medicine, Mahidol University, Bangkok 10400, Thailand; 3 Centre for Tropical Medicine, Nuffield Department of Clinical Medicine, University of Oxford, Oxford OX3 7LJ, UK; 4 The Ethox Centre, Department of Public Health, University of Oxford, Oxford, England, UK; 5 Global Health Bioethics Network, Oxford, England, UK; 6 Faculty of Arts and Social Science, Universiti Tunku Adbul Rahman, Kampar, Malaysia

**Keywords:** Ethics, Evaluation, Community engagement, Community advisory boards, Developing countries, Thailand, Myanmar, Global health, International research

## Abstract

**Background:**

Community engagement is increasingly promoted as a marker of good, ethical practice in the context of international collaborative research in low-income countries. There is, however, no widely agreed definition of community engagement or of approaches adopted. Justifications given for its use also vary. Community engagement is, for example, variously seen to be of value in: the development of more effective and appropriate consent processes; improved understanding of the aims and forms of research; higher recruitment rates; the identification of important ethical issues; the building of better relationships between the community and researchers; the obtaining of community permission to approach potential research participants; and, the provision of better health care. Despite these diverse and potentially competing claims made for the importance of community engagement, there is very little published evidence on effective models of engagement or their evaluation.

**Methods:**

In this paper, drawing upon interviews with the members of a Community Advisory Board on the Thai-Myanmar border, we describe and critically reflect upon an approach to community engagement which was developed in the context of international collaborative research in the border region.

**Results and conclusions:**

Drawing on our analysis, we identify a number of considerations relevant to the development of an approach to evaluating community engagement in this complex research setting. The paper also identifies a range of important ways in which the Community Advisory Board is in practice understood by its members (and perhaps by community members beyond this) to have morally significant roles and responsibilities beyond those usually associated with the successful and appropriate conduct of research.

## Background

Community engagement is increasingly promoted as a marker of good, ethical practice in the context of international collaborative research in low-income countries. [1,2]. There is however, no widely agreed definition of community engagement and approaches adopted and the justifications given for its use vary. In addition to its agreed *intrinsic* value as way of treating communities with appropriate respect, community engagement is also often taken to be of *instrumental* value in many different ways. Community engagement is, for example, seen to be of value in: the development of more effective and appropriate consent processes; improved understanding of the aims and forms of research; higher recruitment rates; the identification of important ethical issues; the building of better relationships between the community and researchers; the obtaining of community permission to approach potential research participants; and even in the provision of better health care. Despite these diverse and competing claims made for the importance of community engagement, there is very little published evidence on effective models of engagement or their evaluation. In this paper, we describe and critically reflect upon an approach to community engagement developed in the context of international collaborative research on the Thai-Myanmar border and report on an attempt to develop an approach to evaluation for use in this complex research setting.

### Research on the Thai-Myanmar border

Since 1986, the Shoklo Malaria Research Unit (SMRU) has provided basic healthcare and carried out research aimed at reducing the impact of multi-drug resistant malaria and other infectious diseases in the area on the border between Thailand and Myanmar. SMRU’s main area of interest is in malaria, with special interest in children and pregnant women – the groups most at risk from malaria – and it has combined this research with the operation of antenatal clinics along the border. SMRU currently runs multiple free clinics for the Karen and Burman border population that would otherwise lack access to health care. The most distant clinic is about 120 km from the SMRU office in Mae Sot. Carrying out research and providing care along the border presents a range of unique practical and ethical challenges. This is in part because the people who live near the border are mostly migrants or refugees from elsewhere in Myanmar who have, since the 1980s, moved to the border area to escape economic hardship and sometimes conflict and persecution. There are currently thought to be two million migrants from Myanmar living in Thailand (about 150 000 in refugee camps) and a further million ‘internally displaced’ people living near the border inside Myanmar, although numbers are difficult to verify. The vast majority of these people, who come from a very diverse range of different ethnic, religious, political and language groups, live in insecure, unsafe conditions and face a number of serious health difficulties.

Until 1995, SMRU focused its activities primarily in the refugee camps in the border area and a strong collaboration was established with non-governmental organisations (NGOs) to control malaria in the refugee population through the operation of “the Malaria Task Force” (MTF). This was largely successful and malaria is now a minor problem within the camps. As a consequence, whilst SMRU remains responsible for antenatal and obstetric care and the treatment of malaria patients in refugee camps such as Mae La, since 1995 it has increasingly extended its activities to reach out to the wider displaced populations who have little alternative access to malaria diagnosis and treatment or antenatal care. This is done in collaboration with the Thai Ministry of Public Health (MOPH) and in close collaboration with the district hospitals and the Tak provincial health authorities. In 2001-2003 the Tak Malaria Initiative, supported by the Bill & Melinda Gates Foundation deployed the malaria control strategy developed by SMRU in the refugee camps to all 200 affected villages in the province’s border districts with substantial success [3]. When the funding from the Gates Foundation came to an end, the MOPH took over the program.

The border between Thailand and Myanmar is at the forefront of the global fight against malaria because it is on the frontline in the battle against the development and spread of resistance to anti-malarial drugs [4]. Malaria parasites found here are some of the most drug-resistant on earth and their expansion and spread is a very real global threat (research has already demonstrated that the most drug-resistant malaria parasites found in Africa originated in South East Asia). This is particularly urgent and important in the populations living along the border because there is published evidence that the malaria parasites may become tolerant even to the most advanced artemisinin combination therapies (ACTs) now at the forefront of global malaria treatment [5].

### Rationale for the establishment of the community advisory board

The Tak Province Border Community Ethics Advisory Board (T-CAB) was established in January 2009. A brief history of the CAB has been published elsewhere [6]. Its founding document, the T-CAB charter, which is available in English, Thai, Karen and Burmese describes the operational guidelines and constitution of the CAB. At the time of its establishment, the CAB had three main goals [7]. The first of these was that, after a period of training – about diseases such as malaria and the nature and goals of research – its members would be able to advise on whether a study is acceptable to and perceived as beneficial by, the communities in the region. The second was that, the CAB would play a key role in advising researchers on the ethical and operational aspects of proposed studies such as: informed consent procedures, fair compensation, risks and benefits, how to protect the confidentiality of research subjects, and so on. The third aim was that the CAB would act as a means of communication between the communities and researchers. It would on the one hand, provide communities with an opportunity to express views on proposed research and to influence and direct research aims, and on the other, provide a means by which the researchers might feedback the results of the research to the community.

### An example of an issue discussed by the CAB

The CAB has met formally 30 times since its establishment and over this period it has considered and commented on 28 studies. The following is an illustrative example.

A study was proposed, which would involve the recruitment of people, some of whom would be likely to have a glucose-6-phosphate dehydrogenase (G6PD) deficiency: a common hereditary condition that protects against malaria but also predisposes towards haemolysis. The study involved treatment with primaquine (a licensed and widely used antimalarial). Primaquine is usually not recommended for people with G6PD deficiency, but an effective radical cure of Plasmodium vivax malaria (other drugs cannot prevent relapse) was required for this population and so dosages and safety needed to be assessed in a highly controlled environment. Because of the risk that some of those treated would suffer haemolysis the proposal was that standby blood donors would be available in the unlikely event that a blood transfusion was suddenly required.

The T-CAB discussed the risks and benefits of the study and eventually decided that the small risk of emergency transfusion among participants was outweighed by the potentially large benefit to local people if treatment guidelines could be revised to allow an effective drug for vivax malaria to be widely used. Whilst there was in the end consensus about this issue, the requirement for standby blood donors generated intense debate over what could and could not be expected of community members and led to a discussion about whether this crossed a threshold at which payment should be made to compensate for the time and inconvenience demanded. This was the first time compensation for non-study participants - in this case standby blood donors – had been discussed. This led to a wider discussion about benefits and it is hoped that the T-CAB will now play a leading role in drawing up a blanket policy on payments to study participants, to achieve consistent standards between studies. There is a practical concern because various international sponsors of studies in the region have differing policies on remuneration. A community agreed position would help local researchers to insist on consistent guidelines when negotiating study arrangements with sponsors.

### Who are the CAB members?

The 2012 CAB has 12 members who live in a range of different settings in the border area. Seven of them live in villages opposite the SMRU clinics on the Myanmar side of the border and five live on the Thai side. There are nine men and three women on the CAB who are between 26 and 60 years of age. There are currently three NGO workers, three teachers, three farmers, one village officer, a pastor, and a housewife. All CAB members are from Karen or Kachin ethnic groups. Languages spoken include: Karen (two forms), Thai, Burmese, and Kachin. The highest attained educational levels of the CAB members are: primary (3), middle school (1), high school (1), pre-university exam (2), undergraduate (3), Masters (1) monastery education (1), and teacher education (1). CAB members are paid a 500 Bhat (approximately US$ 17) per diem for attending the CAB meetings, which is the standard rate paid by NGOs in the Mae Sot area.

## Methods

### Methodological challenges in evaluating community advisory boards

It was recognised at the outset that, whilst the goals described above were appropriate as a starting point, they would need to develop over time with the growing experience of the CAB members and of SMRU, and in the light of appropriate evaluation to explore whether it is achieving the aims it set itself, whether these aims are appropriate, how the CAB is understood by its members and members of the border communities and so on.

Perhaps surprisingly, however, given its importance in the context of international research ethics, very little has been published on the evaluation of community engagement.

*Engaging vulnerable community stakeholders in medical research is less of a controlled and predictable science than we might wish. Nevertheless, it seems curious that we invest millions of dollars in product development, clinical training, design and building of facilities….. but often leave vital processes of community engagement to trial and error*[7].

Whilst there have recently been some examples of published attempts to share experiences in and models of good practice in community engagement, including a number discussing the establishment of community advisory boards elsewhere [8-14], there remains a dearth of evidence and advice about the development, introduction and evaluation of sustainable community engagement activities and there have been a number of calls for the evaluation of the many different models of engagement [15-19]. Against this background, and because of a strong view that it would be necessary to develop a model of evaluation designed specifically for this rather unique and complex setting, it was decided to carry out a small number of interviews with the existing CAB members to begin the process of developing an approach and an appropriate set of measures against which to evaluate the activities of the CAB.

In the design of the study, careful consideration was given to the question of who should conduct the interviews with CAB members. Ultimately this came down to a question about whether interviews should be conducted by KML, who is the facilitator of CAB meetings, or by MP or PYC through a translator. Each approach offered both advantages and disadvantages. The advantages offered by KML conducting the interviews were that he is known to and respected by the CAB members, has a great deal of experience of working in the border region and has had on-going success in promoting and facilitating open, critical discussion of difficult topics at CAB meetings. The disadvantages were that KML is closely identified with SMRU and there was a concern that this might mean that CAB members would feel unable to speak freely about the role of SMRU in the region. In the end, after much discussion and following observation by MP and PYC of a CAB meeting facilitated by KML the decision was made that these disadvantages were greatly outweighed by the advantages offered by KML’s facilitation skills and the greater challenges of conducting interviews through a translator.

In order to mediate the effects of KML’s established role as much as possible a number of measures were taken. Interviews carried out by KML followed a topic guide developed by CPK, KML, PYC, MP, and FN. Verbatim transcripts of these interviews were then translated into English by KML and PYC. The interview transcripts were read independently by MP, PYC, KML and CPK, and emerging themes were discussed and agreed by all team members. A draft version of the main findings of the interviews was then presented to and discussed with the CAB members by MP and PYC. Individual CAB members were also asked independently about certain issues e.g. about group dynamics. Where it was judged appropriate, modifications were made to the analysis to correct for obvious misunderstandings or misinterpretations.

### The motivations and experiences of CAB members

The focus of the interviews carried out with CAB members was on their views about the appropriate role of the CAB and of its members, the expected benefits of the CAB – for the community, CAB members themselves, and for researchers - and the potential challenges they see as arising in practice with the potential to make the realisation of these benefits less likely.

## Results

In what follows, we set out the key themes emerging from the interviews with CAB members. In the subsequent ‘Discussion’ section we assess the implications of these findings for ways of understanding the role of CABs and for the development of models of effective evaluation.

### Perceived role of the CAB

When asked about how they understood the role of the CAB, CAB members said that they saw the CAB as fulfilling three broad functions. In part they saw its role to be to facilitate communication from SMRU to the community, to ensure that the villagers know about SMRU activities. But they also described the role of the CAB as to ‘act as a bridge between SMRU and the villagers’ in a number of other ways which suggest a more active engagement by the community. They described their role as ‘representing’ the community in a number of different senses including: ensuring that the research carried out responds to genuine community needs i.e. is of ‘social value’; protecting the community from potential harms; approving or rejecting proposed research. One CAB member described the role of the CAB as being to ‘empower’ the community.

In addition to these perceptions of the role of the CAB, which are largely in accord with discussions of CABs in the literature and also with those in the original aims of the CAB as set out in the T-CAB charter, it was striking that a number of CAB members also saw the CAB (and CAB members themselves) as having a third, important, function in providing health care or health education.

My role as a CAB member is to provide health education to my neighbours such as hand-washing, boiling water, sleeping with bednets, taking care of health, going to the clinic and don’t self-medicate. (Female teacher, age 30)

I think we should be doing more education and harm reduction in the community. (Male NGO worker, age 26)

We should visit the villagers, treat villagers, and do education. (Male farmer, age 44)

This striking finding is returned to and discussed further, below.

### Motivations for joining

In addition to their views about the role of the CAB, members were asked about their own personal motivations for joining and why some of them have continued to serve as members (7 of the 12 current CAB members were founding members of the CAB serving their fourth term; all current members have served for more than 2 years). In some cases CAB members had been ‘assigned’ to the CAB by an official committee in their community – perhaps because they had a travel permit that would enable them to attend, or because they spoke several languages - but in most cases members described themselves as having chosen to join the CAB for reasons related to their views about the role of the CAB as described above. That is, they joined the CAB in order to ‘contribute to’ or to ‘benefit’ the community, to ‘represent’ the community or to ‘improve communication’ between the community and SMRU. In addition to these community-oriented reasons for joining the CAB many members also gave more personal reasons. Many of these were related to the perceived opportunities the CAB might offer for ‘learning’ – either about malaria, medical research or health care on the one hand or about other villages and communities in the area on the other. Two members of the CAB also highlighted the value they placed on simply being ‘associated with’ SMRU and the hope that this might lead to increased status both in the community and at SMRU clinics e.g. in access to better care.

A strong factor in nearly all of the CAB members’ responses was the existence of an important moral aspect to their motivation to join the CAB. This was expressed in a number of different forms. For some members this was because they felt ‘a duty of care to the villagers’ or ‘a sense of duty and responsibility’. For others it was about making ‘sure that the research suits our needs and not just those of the SMRU’ or to ensure that ‘research meets community needs’ or feeling a responsibility to ‘represent community views’. And these moral considerations also informed the ways in which CAB members analysed ethical issues. For some this was about ensuring that the community and research participants were ‘protected from harms’ or thinking through the ‘benefits, outcomes and disadvantages’. For one CAB member this was a more imaginative, relational exercise,

I try to imagine my pregnant relatives and speak on their behalf. (Male Health NGO worker, Age 38)

### The benefits of the CAB for CAB members

In order to gain a better understanding of why CAB members joined the CAB and how they understood its functions, we asked them what they considered to be the main benefits of the CAB to CAB members, community members, and to SMRU. When asked about the benefits to CAB members themselves, members identified a surprising range of potential and actual benefits. Many CAB members identified important social benefits of becoming a CAB member including the opportunity to meet people from and learn about other villages and communities in the border area. Such social benefits also included a sense that CAB membership brought with it a greater social status and greater sense of being involved in the community as well as, for one CAB member, increased status in the eyes of SMRU,

They treat me with more respect, otherwise I am just another patient. (Male farmer, age 44)

In addition to these social benefits, some CAB members indicated that CAB membership had brought personal health benefits.

Before I was a member I used to self-medicate, now I complete the treatment. (Male farmer, Age 45)

However, notwithstanding the importance of these two forms of benefit – social benefits and healthcare benefits - perhaps the most important personal benefits for CAB members arising out of their CAB role related to its contribution to what might be called their ‘personal and/or professional development’ in two respects. Firstly, as a place to learn and, secondly, as a catalyst for the taking on of other roles in the community.

### The CAB as a place to learn

There was a widely shared view among CAB members that one of the main benefits of the CAB for them during their time as members had been as a place to learn. When asked about the kinds of areas in which such learning had occurred, CAB members described a range of experiences. For many CAB members the CAB had provided a good opportunity to learn about the other communities living in the border area. In many cases, one of the key areas of learning had been in relation to health and disease regarding their own health care and the kinds of health issues in the wider border area more generally: ‘I have learnt about pregnant women and TB’, ‘I gained knowledge about malaria’. Unsurprisingly perhaps, another important area of learning had been in relation to research and the activities of SMRU itself. For others, this had been the first time they had come in contact with the idea of ethics and more specifically with the ethics of research. And for some, this had been a revelation,

Now I know that researchers can’t just do what they like, they need to know about the community, about ethics and about the guidelines. (Male farmer, Age 38)

For others, this had led to a belief that not all the rumours they had heard before were true,

I had heard that western doctors were trying out drugs on people that should be tested on animals. My involvement in CAB shows this isn’t true. I now know that doctors are bound by ethics. (Male pastor, age 54)

Some CAB members highlighted the ways in which their membership of the CAB had provided them with important skills which might be useful elsewhere in their lives. For example, one CAB member said that one of the most important things she had learnt was how to organize meetings and a relatively younger member of the CAB said CAB membership meant that she could work with older people at the same level whereas she would not normally do so. This leads into the second main way in which CAB members saw their membership as providing important personal benefits.

### CAB membership leading to other roles

Several of the CAB members said that one of the main benefits for them of CAB membership had been the fact that it had led to other roles in the community. These roles fell into two broad two categories. The first of these, echoing earlier comments about the healthcare/health educational role of the CAB, was that CAB members increasingly found themselves taking on roles as health educators and in some cases even as health providers in the community. This was expressed in a number of different ways:

I advise people about malaria. (Female, teacher, Age 23)

I advise them to complete the [antimalarial] treatment. (Female teacher, Age 23)

I explain health-related problems to the villagers. (Male Village Officer, Age 47)

I advise people about treatments to take and about when to come to the clinic. (Male Village Officer, Age 47)

When I see a sick person, I explain what I know and encourage people to go to the clinic. (Male farmer, Age 44)

I assist them and send them to hospital (gives an example of helping a woman to give birth). (Female housewife, Age 48)

In addition to CAB members finding themselves taking on health education or health provider roles in the community, they also found themselves providing other forms of support to the people – mainly refugees and migrant workers – such as helping them with the obtaining of travel or other documentation.

I assist people from my village in Myanmar who don’t have documents. (Female, housewife, 48)

These additional roles were seen in a positive light by CAB members and were often related to a broader sense of personal development,

I’ve changed a lot, it is a positive experience, I gained experience in the community’. (Male, farmer 44)

‘I used to just stay at home, now I go out and educate people’. (Female housewife, 48)

### The benefits of the CAB for the community

It was clear from their comments that CAB members saw the existence of the CAB as beneficial for the community in a number of important ways. They saw it as leading to increased understanding in the community of the research taking place in the area as a consequence of the potential it had to enhance communication between SMRU and the community. CAB members also saw the CAB as offering the potential for other important benefits arising out of the role of the CAB as a ‘bridge between SMRU and the villagers’ in a number of other senses that suggest a more active engagement by the community. As mentioned above, they saw their role and that of the CAB as ‘ensuring that the research carried out responds to genuine community needs’; protecting the community from potential harms; having the opportunity to reject proposed research and, more broadly, as ‘empowering’ the community. Consistent with their view that the role of the CAB also included the provision of healthcare and of health information, CAB members also identified these as important ways in which the CAB benefits the community.

### The benefits of the CAB for SMRU

What about the benefits of the CAB for SMRU? When asked about this, CAB members said that the primary benefit of the CAB to SMRU is that it provides a ‘trusted bridge’ between SMRU and the community. When asked what this meant in practice, they said that the CAB could act as a source of information and advice about the communities in the border area and their ‘views, beliefs, customs and taboos’. They also said that one role of the CAB would be to offer practical solutions to specific practical problems such as the appropriate wording of consent forms and so on. One CAB member also said that the CAB had been useful to SMRU in situations where there had been a need to interpret and adapt international guidelines relating to assent in children to local conditions and practices.

### Challenges and limitations

Having explored the roles and potential benefits of the CAB, members were then asked about the limitations of the CAB as a model of community engagement and the challenges to its effective functioning in the context of the Thai-Myanmar border area. The challenges they identified were of five broad forms: challenges arising out of the local geopolitical context; language and communication; issues relating to the independence of the CAB; interpersonal challenges in CAB meetings; and, the low levels of awareness of the CAB in the communities.

### Geopolitical context

As outlined in the introduction to this paper, the Thai-Myanmar border presents a number of geographical, political and cultural challenges and unsurprisingly, these are also factors in the effectiveness of the CAB. CAB members need to travel long distances across difficult terrain to attend meetings. They face a number of risks and dangers in doing so including the risk of being arrested and, in some cases, risks of violence. CAB members expressed particular concern about dangers for women travelling alone. In general, CAB members thought that the focus of the CAB on health matters offered a certain amount of protection but the political instability in the region was a concern to all.

### Language, communication and culture

The CAB members, and the scientists who present their research to them, speak a range of different languages. Whilst some CAB members speak or are able to understand more than one language, there is no single common language and so multiple translations are a key feature of discussions at CAB meetings. Given this, it is not surprising that communication and the need for continuous translation were seen as challenges by CAB members. However, this was seen as much less of a problem than might be expected. Living as they do in a multi-lingual area, CAB members were mostly very pragmatic in the face of these linguistic differences and felt that meetings were nonetheless effective. More than one CAB member expressed the view that ‘language is less important than culture’ indicating that cultural differences between CAB members were more of a challenge. Some CAB members wondered whether it might be more effective to set up ‘cluster’ CABs in the different communities to counter this set of challenges but most seem to view language as less of a problem than might have been expected.

### Independence and dependence of the CAB

Whilst highlighting the importance of the ‘independence’ of the CAB and of the CAB members, CAB members also recognized the key role played by SMRU in the establishment and in the sustainability of the CAB both in practical terms through the provision of transport, meeting rooms and so on, but also in terms of facilitation and leadership of discussion in what is a diverse and low-income setting. They also mentioned the importance of SMRU as a key health provider as a factor which means that there are inevitably limitations to the degree to which the CAB might be thought of as independent. Overall, the CAB members did not seem greatly troubled by this, recognizing that in the circumstances such role ambiguities were inevitable. They also emphasized the shared interests of SMRU and the CAB regarding the role of SMRU in working for the benefit of the community. Interestingly, some of the CAB members felt that in addition to their relationship with the community, SMRU had a special duty of care to CAB members.

### Interpersonal challenges

A fourth category of challenge to the effective working of the CAB identified by CAB members related to a range of interconnected social or cultural factors affecting the possibility of free and open debate of ethical issues. Some of these issues were to do with the different social status of CAB members, which inevitably, even if unintentional, created a hierarchy which affected some members’ willingness or ability to contribute. CAB members also reported that in addition to issues relating to hierarchy, cultural views about the legitimacy of public disagreement, about ‘being wrong’, and, about ‘what others might think’ also sometimes made it difficult for free and critical discussion to take place. Most members did not feel that there were any gender issues.

If they speak first I don’t want to disagree. (Male, NGO staff, 38)

Sometimes I don’t speak because I’m afraid I might be wrong. (Female, teacher, 30)

Many CAB members placed a great deal of importance and value on the facilitation role of a member of SMRU staff – himself, interestingly, a Myanmar doctor – which they believed tended to mediate some of these cultural barriers.

### Low levels of awareness of the CAB in the community

Finally, it became apparent during the interviews that an important limitation on the effectiveness of the CAB – insofar as its effectiveness is linked to the involvement of the wider community – was the fact that few CAB members discussed their CAB membership or role with more than a very small subset of community members. When asked specifically about this, many CAB members said that the level of awareness was low. When asked who they personally had told, it became apparent that this was the case.

I told my boss but didn’t tell other people. (Female teacher, Age 23)

I don’t think it is important to tell people. (Male NGO worker, Age 26)

I have not told anyone about my membership. (Female Housewife, Age 48)

I have only told my neighbours. (Female teacher, Age 30)

It is best not to talk – I am not allowed to cross the border. (Female teacher, Age 43)

There were, however, some CAB members who believed there to be a greater level of awareness.

The community knows about my involvement, I act as a middleman. (Male farmer, Age 45)

The villagers are very positive and appreciative. (Male farmer, Age 44)

I have told friends and at township meetings. (Male Village Officer, Age 47)

## Discussion

Our aim in carrying out the interviews reported here was to begin the process of working towards a better understanding of the factors likely to be of importance in the development of an appropriate model for the evaluation of the effectiveness of the T-CAB. We decided to do this partly because of our awareness that there is relatively little published data on the evaluation of community engagement in the context of medical research, but also partly because it was clear to us even at an early stage that it would be necessary for the development of any adequate approach to evaluation in this setting to be informed by some initial pilot work to map out the factors likely to be relevant in this rather unique context. Our analysis has identified a number of factors CAB members identified likely to be relevant to the effectiveness of the CAB.

It is clear that any meaningful evaluation will need to take account of the context in which the CAB is operating and in which the research is to be carried out and the fact that against this backdrop an effective CAB will inevitably be an exercise characterized by flexibility and pragmatic judgement. It is also important to bear in mind that any model for evaluation will need, in addition to the views of CAB members, to take account of input from other key stakeholders such as researchers, research participants and members of communities. The question of input from community members highlights the important role that will need to be played in the development of an adequate model of evaluation by a critical and empirically-driven consideration of how exactly the ‘community’ and ‘communities’ are to be understood in this context.

In addition to these factors, it also became apparent as the process of analysis progressed that the ways in which role and social function of the CAB was understood by its members were much richer than was expected at the outset, and was in many important, and potentially significant ways different to the way in which it is understood by the staff of SMRU or expressed in the T-CAB charter. A powerful and striking example of this was the way in which a core function of the CAB was seen by many of its members as to be to do with providing them with scope for personal development through the opportunities for learning it provided and the ways in which it allowed CAB members to take on new social roles. It is clear that this presents both opportunities and important challenges. A good example of this is the evidence that emerges about the CAB members playing a health educational role e.g. some members provide simple health advice to the villagers, and even contact SMRU clinic staff directly when there is a medical emergency in their village. The potential challenges of this are obvious given that the majority of CAB members have had no formal training in health or health education. However, this finding also suggests that under certain conditions CAB members might be interested in engaging in other ways with the wider communities in the region and, that with appropriate training and support, this might provide an important and innovative approach to the development of community engagement appropriate to complex and widely distributed communities such as those along the border region combining the community advisory board with a range of other outreach activities.

It was also apparent from the interviews that issues of hierarchy, cultural views and age sometimes made it difficult for CAB members to freely give their opinions in meetings. When the CAB was established, a decision was made that there whilst there would need to be a CAB secretary no other formal ‘offices’ would be established in order to attempt to create an environment, at least in the meeting room, where – insofar as this was possible - everyone was equal. The concern was that were a ‘chair’ to be created, the most influential members would be elected and other members would be unable to express their own views. The findings from our interviews suggest that whilst the CAB has worked reasonably well, issues related to hierarchy have influenced levels of participation. It is also our view that other related challenges exist within the CAB which were not expressed in the interviews. One of the more important of these is the political affiliations and inclinations of the members. In the experience of the CAB facilitator, gender did not tend to play a major role when it came to voicing opinions, reflecting the nature of the Myanmar community. However, he did acknowledge, that in the first year of the establishment of the CAB, the female members had tended to keep to themselves only becoming more vocal as time went by. The current CAB has two active long serving female members, both of them primary school teachers.

SMRU has been engaging the community for many years by means of ongoing dialogue and consultation with the community leaders, key workers, patients and their relatives and most importantly, its own staff of which 90% are from the border area. The CAB supplements this existing and ongoing consultation. Although the CAB model was chosen as a way of formalising community engagement, it is not the conventional CAB model, where a CAB is established for a particular study or programme e.g. HIV vaccine study, for a fixed length of time in a defined geographical area where the community members are somewhat homogenous, at least for the purpose of the said study or programme, and members are somewhat representative of the community. Instead, the non-conventional T-CAB which seemed to have been established against all odds, consist of members who live along the porous Thai-Myanmar border, where the population is fluid and comprised of many overlapping sub-communities and the members review a wide range of projects.

## Conclusions

In this paper we describe the findings of interviews exploring the motivations and perceptions of members of the Tak Province Community Advisory Board on the Thai-Myanmar border and their views about the role of the CAB and potential challenges to its effectiveness. This was a small scale preliminary study intended as an initial attempt to identify the factors identified by one key group of stakeholders as relevant to the development of an appropriate model for the evaluation of the CAB. A number of important factors were identified. Clearly, any final approach to the evaluation of the community advisory board will also need to be informed by other key stakeholders such as researchers, research ethics committee members, and members of the wider communities in the border area, but the findings of this study have, however, nevertheless provided a number of interesting and potentially important insights into the ways in which this community advisory board is understood by its members and suggests that it is a much more complex social phenomenon than expected. Whilst this was intended as an attempt to take a first step towards a model of evaluation, it has also become clear that more work needs to be done to map out the complexities of the community advisory board as an innovation, and its potential as an intervention in research ethics.

## Competing interests

The authors declare that they have no competing interest.

## Authors’ contributions

FN, NJW, NPJD, PYC, KML and MP designed the strategy for community engagement. Interviews with CAB members were carried out by KML following a ‘topic guide’ developed by CPK, KML, PYC, MP, and FN. Verbatim transcripts of these interviews were then translated into English by KML and PYC. Each of these transcripts was read by MP, PYC, KML and CPK. Emerging themes were discussed and agreed by all team members. A draft version of the main findings of the interviews was presented to the CAB members by MP, KML and PYC and members were also asked independently about certain issues e.g. about group dynamics. MP, KML and PYC wrote the first draft of the paper. FN, ND, NW commented on and made significant contributions to subsequent drafts to the final version, which was then agreed by all authors. All authors read and approved the final manuscript.

## Authors’ information

Khin Maung Lwin and Phaik Yeong Cheah are joint first author.

## Pre-publication history

The pre-publication history for this paper can be accessed here:

http://www.biomedcentral.com/1472-6939/15/12/prepub
